# Counting crows: population structure and group size variation in an urban population of crows

**DOI:** 10.1093/beheco/ary157

**Published:** 2018-12-08

**Authors:** Florian Uhl, Max Ringler, Rachael Miller, Sarah A Deventer, Thomas Bugnyar, Christine Schwab

**Affiliations:** 1 Department of Cognitive Biology, University of Vienna, Vienna, Austria; 2 Department of Ecology and Evolutionary Biology, University of California Los Angeles, Los Angeles, CA, USA; 3 Department of Integrative Zoology, University of Vienna, Vienna, Austria; 4 Department of Psychology, University of Cambridge, Cambridge, UK

**Keywords:** *Corvus corone*, crows, fission, fusion dynamics, group size, population structure

## Abstract

Social complexity arises from the formation of social relationships like social bonds and dominance hierarchies. In turn, these aspects may be affected by the degree of fission–fusion dynamics, i.e., changes in group size and composition over time. Whilst fission–fusion dynamics has been studied in mammals, birds have received comparably little attention, despite some species having equally complex social lives. Here, we investigated the influence of environmental factors on aspects of fission–fusion dynamics in a free-ranging population of carrion and hooded crows (*Corvus corone ssp*.) in the urban zoo of Vienna, Austria over a 1-year period. We investigated 1) the size and 2) spatio-temporal structure of the local flock, and 3) environmental influences on local flock and subgroup size. The local flock size varied considerably over the year, with fewest birds being present during the breeding season. The spatio-temporal structure of the local flock showed 4 distinct presence categories, of which the proportions changed significantly throughout the year. Environmental effects on both local flock and subgroup size were time of day, season, temperature, and weather, with additional pronounced effects of the structure of the surroundings and age class on subgroup size. Our findings show environmental influences on party size at the local flock and subgroup level, as well as indications of structured party composition in respect to the 4 presence categories. These results suggest that environmental factors have significant effects on fission–fusion dynamics in free-ranging crows, thereby influencing social complexity.

## Teaser

With data collected over a 1-year period, we detail the spatio-temporal structure of a flock of crows displaying distinct social categories. Presence of carrion and hooded crows was affected by the environmental factors that modulate the party size component of fission–fusion dynamics and thereby influence social complexity.

## INTRODUCTION

Living in social groups can facilitate predator protection and enhance foraging opportunities, though it may also increase food competition and social complexity (Krause and Ruxton 2002). The benefits of group living typically correspond with an increase in group size, for instance, as more individuals are more likely to spot a predator (Hamilton 1971; Treisman 1975). Social complexity arises mainly due to the formation of social relationships like social bonds and dominance relations (Harcourt and de Waal 1992; Cords and Aureli 2000), which in turn help individuals to cope with competition (Scheiber et al. 2005; Smith et al. 2008). In societies structured by social relationships, the number and spatio-temporal distribution of potential interaction partners may further contribute to a species’ level of social complexity (Kappeler and van Schaik 2002).

Fission–fusion dynamics—changes in group size and composition over time (Kummer 1971)—affects the ratio and likelihood of meeting particular individuals. These dynamics may enhance certain cognitive skills, like impulse control and inferential reasoning, as individuals that have been away from the group for a period of time may need to readjust to new situations, like changes in the dominance rank hierarchy or alliances (Paz-y-Mino et al. 2004; Amici et al. 2008; Aureli et al. 2008). Following the introduction of the term, research on fission–fusion dynamics has focused mainly on mammals (Aureli et al. 2008) and comparatively few studies have addressed such dynamics in birds (Silk et al. 2014). This is surprising as many bird species show high variation in group size and composition, particularly outside the breeding season (Clayton and Emery 2007).

The organization of avian groups is highly variable from individuals living in pairs to those living in family groups and communally in large mixed-sex and age groups (Bond et al. 2003). In most systems, however, male–female pairs represent the key social unit (also termed primary relationship) (Cockburn 2006; Boucherie et al. 2016), as pair partners cooperate for reproduction, but also to gain/maintain dominance status and/or access resources (Scheiber et al. 2005; Emery et al. 2007). A recent study on adult rooks found that, in addition to pair bonds, individuals have secondary relationships with other colony members, suggesting that larger corvid groups are structured by different social layers (Boucherie et al. 2016). Similar patterns have been proposed for geese (barnacle geese, *Branta leucopsis*, Kurvers et al. 2013; greylag geese, *Anser anser*, Scheiber et al. 2013) and parrots (spectacled parrotlets, *Forups conspicillatus*, Wanker 1999; review in Bradbury and Balsby 2016). A refined social structure can also be seen in family units of cooperatively breeding corvid species (Brown 1970; Woolfenden and Fitzpatrick 1984; Baglione et al. 2002) and, to some extent, even in nonbreeder flocks. Raven nonbreeders, for instance, show different degrees of vagrancy (Braun et al. 2012; Loretto et al. 2017), whereby birds with low vagrancy status (“residents”) engage in sophisticated interactions, including third-party interventions in others’ conflicts (Szipl et al. 2017) and bonding attempts (Massen et al. 2014). Taken together, it appears that the social system of some avian species is more complex than simply brief aggregation at shared resources (Vander Wall and Balda 1977). Rather, these avian systems are characterized by individualized membership and the formation of social relationships outside of the breeding pair.

Exploration of the ecological factors affecting group formation, specifically its composition and size, can be informative for studies on fission–fusion dynamics. Studies on white-throated magpie-jays (*Caloditta formosa*), for instance, showed a positive relationship between food availability and group size (Langen and Vehrencamp 1998). Similar effects were also found in primates (Chapman et al. 1995; Chapman and Pavelka 2005), and in lions (*Pantera leo*, Caraco and Wolf 1975). Another ecological factor that can influence group size is the openness of the habitat, for example, the presence of larger groups in more open habitats (Peek et al. 1974; Thirgood 1996). However, surprisingly little is known about the impact of environmental factors on the grouping behavior of opportunistic corvids like carrion crows (*Corvus corone*).

The present study focused on a population of wild, free-ranging carrion crows utilizing the area of Vienna Zoo (Tiergarten Schönbrunn) in Vienna, Austria (hereafter “local flock”). Carrion crows are highly opportunistic in terms of foraging and habitat use (Coombs 1978; von Blotzheim 1993). To our knowledge, carrion crows and common ravens do not differ significantly in their social structure: individuals aggregate at food sources and groups show hierarchies determined by age, sex, and body size (Heinrich 1989; Richner 1989a; Braun and Bugnyar 2012). In contrast to ravens (Braun et al. 2012; Loretto et al. 2015) and American crows (Stouffer and Caccamise 1991), fission–fusion dynamics have not yet been explored in carrion crows.

The aim of our study was to examine key aspects of fission–fusion dynamics in this focal population of crows. According to the framework proposed by Aureli et al. (2008), the degree of fission–fusion dynamics in a species can be determined via 3 components: variation in party size, party composition, and spatial cohesion. Here, we draw on this framework and used it as a heuristic device to establish whether crows at Vienna Zoo show temporal variation in social structure across time. To make the distinction between Aureli et al.’s framework and our measurements clear, we use the term “party” only when referring to components of the framework itself and use different expressions when referring to our own empirical measures. Specifically, we investigated variation in party size by examining changes in the number of crows in the zoo area, which we refer to as “local flock size,” and the number of birds foraging together, which we refer to as “subgroup size.” These two measures have also been used for describing party size in ravens from a global and local perspective (Braun and Bugnyar 2012), i.e., whether birds join a flock in a specific area (e.g., zoo) and whether they join particular foraging groups within this area (e.g., at particular enclosures). Furthermore, we took a first step towards investigating the variation in party composition at the level of the local flock by looking at changes in the crow’s residency status (categories due to presence/absence patterns) across seasons. Note that we did not focus on variation in the identity of individuals within a subgroup, which would be expected when mapping the framework of Aureli et al. (2008) directly onto the crow social system. We hypothesized that the local flock 1) varies over the year in size and 2) has a nonrandom spatio-temporal social structure, i.e., the residency status of individuals follows specific patterns. Environmental factors likely influence the size of 3) the local flock and 4) the subgroups of crows present at the zoo.

Our predictions were: 1) more crows would be present in the zoo outside the breeding season, as territorial pairs would fend off nonbreeding birds, and offspring of the breeders would still be present after the breeding season (Schwab, personal observation). 2) The local flock would be structured as local birds, likely territorial breeders, and nonbreeders with different degrees of vagrancy, which resembles the social organization of ravens (Heinrich et al. 1994; Braun and Bugnyar 2012) and show a similar ecology to American crows (Marzluff and Angell 2005). Notably, the residency/vagrancy status of individual crows could vary across the year, hinting towards changes in flock composition. 3) The size of the local flock would be influenced by key environmental factors. Weather would influence the local flock size. Fewer birds would be present in rainy weather conditions as nonresident crows would be less likely to fly to the zoo. Alternatively, the crows would be more likely to visit the zoo during rainy weather due to increased food security, as the food is provided for the zoo animals irrespective of the weather. The time of the day would affect the local flock size, as more individuals may arrive when certain zoo animals are being fed, in order to exploit this high-quality food source, such as meat to the carnivores. A higher number of human visitors would increase local flock size as more food becomes available when visitors accidentally drop food or actively feed the birds. 4) Subgroup size would vary with overall local flock size. However, factors relating to the immediate surroundings of the birds (forestation, type of enclosure/visitor area) would also play an important role. Specifically, larger subgroups would be expected in larger open areas for predator protection (Jarman 1974) and in areas with more widely dispersed food, as the feeding competition is lower in such locations (Asensio et al. 2008). Additionally, we expected to find an influence of age on subgroup composition with younger birds showing a stronger tendency to form groups than adult birds, as younger birds are more likely to be part of nonbreeder flocks (Richner 1989b).

## METHODS

### Study site and population

**Figure 1:**
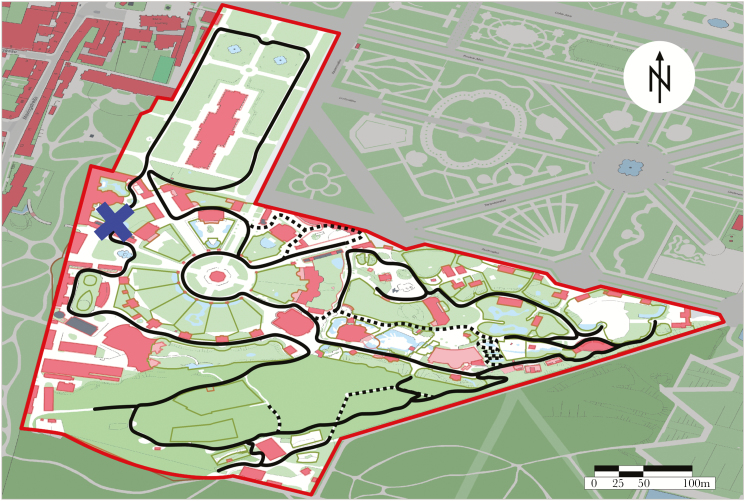
The study area within Vienna Zoo is outlined in red. The black line represents the observation transect and the blue cross indicates the starting point. Dotted lines show temporal deviations when the regular transect was not accessible due to construction work and/or hazardous weather conditions. At dead ends in transects, we only recorded crows in one travel direction.

The present study was conducted at Vienna Zoo (Tiergarten Schönbrunn) (48°10′54.6′′N 16°18′14.3′′E) in Austria (see Figure 1), which is located within the city limits. The Zoo consists of parkland and a forested area. Since 2010, crows in the zoo have been caught using “ladder” and Larsen-traps (Kirchmeir et al., in preparation), and individually color-ringed for identification before being immediately released at the site of capture. At the onset of the present study in 2014, 297 crows were ringed, which rose to 322 by the end of the study in 2015. Of these marked individuals, 129 were sighted again follow release during the duration of the present study. Permission for catching and marking the birds was obtained from the municipal authorities of Vienna (Magistrat der Stadt Wien: MA 22–425/2011/6) and the Austrian Ministry for Science and Research (BMWF-66.006/0009-II/3b/2012).

The study area is ideal for conducting behavioral observations on group formation and dynamics as the crows use several parts of the zoo for foraging on a wide variety of food (Miller et al. 2014; Deventer et al. 2016), are easy to spot and are well habituated to human presence. Vienna lies within a hybrid overlapping zone with presence of both carrion and hooded crows, which as classed as subspecies (*Corvus corone corone* and *Corvus corone cornix*, von Blotzheim 1993; de Knijff 2014) and regularly interbreed (Randler 2008). Hence, the local flock consists of individuals of both subspecies and their hybrids. The hybridization is interesting under genetical and evolutionary aspects because there is still substantial gene-flow between the 2 subspecies (Poelstra et al. 2014) despite evidence for conspecific assortative mating (Risch and Andersen 1998; Randler 2007). In the area around Vienna, the 2 subspecies interbreed regularly (Randler 2008).

The subspecies of each marked individual was visually assessed and recorded during handling when the birds were caught. However, it was difficult to reliably visually identify the subspecies of some individuals during observations in the field, due to challenging environmental conditions, including partly obstructed bodies or variations in lighting. This difficulty is due to some hybrid birds showing similarities to one subspecies (e.g., having greyish plumage parts) over the other, which could lead to mistakes in identifying these individuals in the field (e.g., as a hooded crow rather than a hybrid). We therefore refrained from including subspecies as a factor in our analysis.

### Data collection

We monitored the crows during daylight hours from 8 January 2014 to 31 January 2015, in order to avoid any seasonal bias and observe changes occurring across an entire year (cf. Marra et al. 2015). In winter (8 January to 17 April and 17 October 2014 to 31 January 2015), we conducted 2 observational sessions (“transects” hereafter): one 2-h transect between 0900 and 1300 h (“Morning”) and one 2-h transect between 1300 and 1600 h (“Afternoon”). When daylight hours increased (23 April 2014 to 14 October 2014), we increased to three 2-h transects (per day) between 0800–1200 h (“Morning”), 1200–1500 h (“Noon”), and 1500–1900 h (“Afternoon”). Minor fluctuations in transect duration occurred due to the varying time needed to enter data. Between 27 January–9 February and 10–16 March 2014, no surveys were conducted due to observer illness. We collected data via scan observations along a fixed transect covering the entire zoo (see Figure 1); which took on average 2 h. Occasionally, we had to make minor changes to the transect when some paths were closed temporarily. While we kept the starting point of the transect constant, the travel direction was alternated (clockwise/counter-clockwise).

We entered each observation of a crow, marked or unmarked, into a digital map of the zoo using a GPS enabled pocket PC (MobileMapper 10, SpectraPrecision) with a mobile GIS software (ArcPad 10.2, ESRI). Data for the background map were obtained from the Municipal Authorities of Vienna (MA41) and Vienna Zoo. With each observation, we recorded individual parameters of the bird (age class, ID if individual was marked) as well as behavioral and environmental parameters (Supplementary Table S1). Regarding age class, we differentiated juveniles (birds in their first summer) from older birds (subadults and adults) as the very distinct appearance of juveniles (slender silhouette, begging behavior) could be reliably determined even under difficult lighting conditions. We refrained from using color-based features (von Blotzheim 1993) as these aid in differentiating age classes under good lighting conditions only. We excluded sex because we could not reliably visually determine the sex of unmarked birds in the field. In addition, we recorded the observed subgroup size for individuals seen within spatially associated clusters. We defined subgroups as aggregations of nonflying individuals with a nearest neighbor with a direct line of sight within 5 m (Smolker et al. 1992; Wolf et al. 2007; Hobson et al. 2014). Subgroups were rarely spread across more than one enclosure as most enclosures were separated by physical barriers (trees/walls). We performed a total of 271 transects (104 Morning, 60 Noon, 107 Afternoon), which took place on 122 days (2–3 days a week) with an average of 2.22 (SD: ±0.67) transects per day. These observations were carried out based on observer availability.

### Analysis

Spatial data were analyzed in the GIS software ArcGIS 10.0 (ESRI) and statistical analyses were performed in R Version 3.4.2 (R Core Team 2017). We obtained information on structural characteristics at observation locations via spatial joins from the background map in ArcGIS 10. We characterized and partitioned the study area using the following parameters: openness of the area (“Forested Area”: in forest – out of forest), availability of man-made structures (“On Building”: on building – off building), and type of food found in an area (“Food”: grass, vegetarian, mixed, mainly meat, human gastronomical area) (listed in Supplementary Table S2).

We grouped the data into 3 seasons of equal duration according to the birds’ breeding ecology: the breeding season (February – May), the parental care season (June – September) and the nonbreeder season (October – January). The breeding season lasts from nest building by the territorial breeding pairs until fledging of the chicks. The parental care season starts with the formation of larger groups of juvenile birds with their parents and ends when juveniles become independent from their parents. At this point, the nonbreeder season starts, which ends with the start of the new breeding season.

In order to investigate the changes in the size of the local flock, we calculated a conservative minimum estimate for the local flock size based on the total crow observations during a day (prediction 1). As we could not reliably identify unmarked crows, it is possible that we repeatedly registered unmarked individuals during an observational session. We therefore calculated the ratio of resightings of marked birds as a correction factor (CF) to correct for resightings of unmarked birds as

$$CF=\frac{CM_{t}-CM_{ind}}{CM_{t}}$$ (1)

where CM_t_ is the total number of sightings of marked birds including resightings and CM_ind_ is the absolute number of unique individuals seen that day.

Using this correction factor, we then calculated a local flock size estimate as

$$FS_{e}=CM_{ind}+\frac{CU-CU*CF}{T}$$ (2)

where FS_e_ is the local flock size estimate per day, CU is the total number of sightings of unmarked crows including potential resightings, and T is the number of transects on a given day to account for sampling effort (only used for unmarked individuals as we know the exact number of unique marked individuals seen per day). These data are used for graphical representation as well as the analysis of temporal autocorrelation. For our model calculations on local flock size, we used the flock size estimate per transect (i.e. the total number of crows per transect times the correction factor of the day). We chose this method in favor of capture–recapture calculations (Pradel et al. 1997; Thomas et al. 2002) as we wanted to use our count data with adjustments for potential resightings as a conservative measurement for our models.

We conducted a cluster analysis on the presence–absence data of marked, individually identifiable crows in the zoo to assess the spatio-temporal structure (hereafter: “presence categories”) for the local flock (prediction 2). The parameters included in the analysis were: the number of days an individual was seen, the longest period in days without observation of an individual, and the standard deviation for these periods without observations of an individual. For this analysis, we only used marked individuals that were observed on 5 days or more (*N* = 82), the others (*N* = 47) were classified as rare visitors. We calculated Euclidean distance matrices for the standardized values of these variables and used hierarchical clustering (hclust, method: complete, package: stats). We chose clusters that were well formed and showed long branches to determine the presence categories. To investigate the spatio-temporal structure, and in particular, whether the presence and absence of individuals was significantly different between categories and seasons (prediction 3), we then compared the percentage of unique individuals per day for each presence category between seasons by calculating Friedman tests and 2-tailed paired Wilcoxon tests using a nonparametric bootstrap (sample size 38 and 10,000 iterations). We also applied a Bonferroni correction (α = 0.017) for multiple testing. We calculated the relative number of days that birds of each presence category (days seen/total days with observations) were seen between seasons using approximative Friedman tests based on Monte-Carlo resamplings (10,000) and exact paired Wilcoxon signed-rank tests in the coin package (Hothorn et al. 2008) with Bonferroni correction (α = 0.017). Lastly, we compared the group sizes between the different presence categories by calculating Kruskal–Wallis and pairwise post-hoc 2-tailed Mann–Whitney *U* tests using Bonferroni correction (α = 0.008).

We used generalized linear mixed models to evaluate the impact of environmental factors on both local flock size and subgroup size of unmarked and marked birds (predictions 3 and 4). For the analysis of the local flock size, the response variable was the estimate of the local flock size per transect, in order to include the effect of transect time into the model. The full model included date as a random factor and the following fixed factors: weather, temperature, transect time, season, visitors for both flock size estimate and subgroup size, age class, forested area, food, and on building (for the respective levels of the fixed factors, see Supplementary Tables S1 and S2). The response variable for the latter subgroup-size models was the subgroup size minus one to fit the negative binomial distribution for the model. The full model included date as a random factor and the following fixed factors: weather, temperature, observational session, season, number of visitors, number of crows present during a session, age class, forested area, predation risk, food in enclosure, on building and area (for the respective levels of the fixed factors, see Supplementary Tables S1 and S2).

We calculated all possible models (32 for the local flock size estimate and 1024 for the subgroup size) via lme4 (Bates et al., 2015) in MuMIn (Barton 2016) and used Akaike’s Information Criterion for model selection (Anderson and Burnham 2002; Burnham and Anderson 2004). We formed model averages from the weighted estimates of all models (Anderson 2008). We also tested the intercorrelation between the factors for both models (Supplementary Tables S3 and S4) and for temporal autocorrelation (Supplementary Table S5).

## RESULTS

### Spatio-temporal structure of the local population of crows

**Figure 2:**
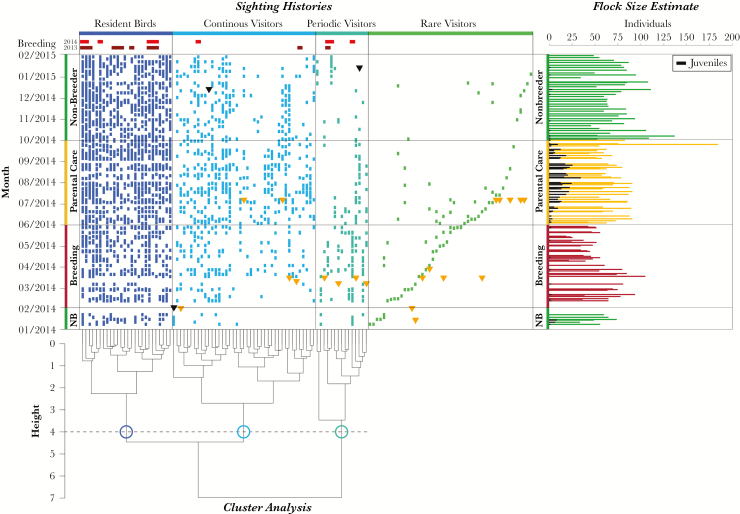
Sighting histories of marked birds in the zoo. The 3 categories determined by the cluster analysis are shown in different colors. The order of the individuals corresponds to the leaves of the dendrogram for the cluster analysis. Rare visitors were not included in the cluster analysis and are ordered in this plot by order of first sighting. The individuals with available breeding related data are denoted at the top of the graph (dark red = 2013, bright red = 2014). Inverted triangles indicate when birds either died (black) or were newly ringed (orange). The flock size estimates per day of observation are shown in the right graph. The colors correspond to the different seasons. This graph also shows the proportion of juvenile birds in the study area designated in black.

In total, we obtained 17,645 observations of marked and unmarked crows between 8 January 2014 and 31 January 2015. The daily estimated minimum zoo population size was 65.2 ± 23.2 crows (mean ± SD) across the 122 days of monitoring, of which 64% (41.4 ± 23.1) were unmarked individuals (Figure 2). The correction factor (CF) found was on average 0.174 ± 0.1 (mean ± SD).

Of 322 marked crows, 129 individuals (40%) were observed at the zoo during the study period. Of these 129 birds, 81 were males (63%), 40 were females (31%), and 8 were of unknown sex (6%). 13 were juveniles in their first year (10%) and 116 were older than a year (90%), as indicated by the color of the inner beak (von Blotzheim 1993).

Not all observed marked birds could be identified (2894 out of 3356 sightings; 86.2% identification rate) as some birds had lost rings, and sometimes lighting and environmental conditions, such as high grass, foliage and observation distance, prevented identification. We omitted these observations in the analysis. The resighting rate varied widely among the individuals, ranging from 67.2% (sighted on 82 out of 122 days) to 0.8% of all observational days (sighted on 1 day only).

The organization of the local flock was mostly in line with our prediction (2), in that it was similar to the social organization of ravens, with local birds and nonbreeders with differing degrees of vagrancy (Heinrich et al. 1994; Braun and Bugnyar 2012). However, we found evidence for one further category in the crows as the result of our cluster analysis partitioned the birds into 3 well defined clusters—presence categories—representing “resident” birds (*N* = 26), “continuous” visitors (*N* = 41), and “periodic” visitors (*N* = 15) (Supplementary Table S6). Crows with fewer than 5 observational days (*N* = 47) were a priori considered as “rare” visitors. The temporal occurrence of the birds at the Zoo is shown in Figure 2, which also shows breeding information of marked individuals breeding in the Zoo in 2013 and 2014 (*resident* birds: 11/26; *continuous* visitors: 2/41, *periodic* visitors: 3/15).

**Figure 3:**
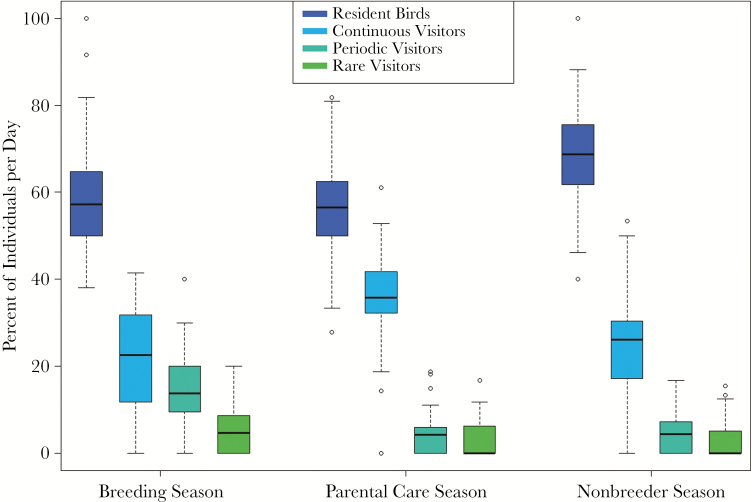
Relative proportions of presence categories per season.

The contribution of crows from all 4 presence categories to the overall population size was significantly different between seasons (Friedman test, nonparametric bootstrap 10,000 iterations, mean ± SE; *resident* birds: *χ*^*2*^ = 15.594 ± 0.064, *N* = 38, *P* = 0.01 ± 0.0004; *continuous* visitors: *χ*^*2*^ = 22.067 ± 0.071, *N* = 38, *P* = 0.001 ± 0.0001; *periodic* visitors: *χ*^*2*^ = 27.925 ± 0.08, *N* = 38, *P* = 0.0002 ± 0.00003; *rare* visitors: *χ*^*2*^ = 6.772, *N* = 38, *P* = 0.144 ± 0.002; Figure 3). The proportion of *resident* birds was significantly higher during nonbreeder season than during parental care season, with the 2 other seasons not differing significantly from one another (Table 1). The proportion of *continuous* visitors was significantly higher during the parental care season than during breeding and nonbreeder seasons, which did not differ significantly from one another. The daily proportion of *periodic* visitors was significantly higher during the breeding season than during the parental care and nonbreeder seasons, with no significant difference between the latter 2 seasons. We found no significant differences for the proportion of *rare* visitors between seasons.

**Table 1:** Pairwise comparisons of the presence categories’ proportions between seasons

	Resident birds	Continuous visitors	Periodic visitors	Rare visitors
BS/PCS	*V* = 387.92 ± 0.676	*V* = 77.65 ± 0.376	*V* = 606.9 ± 0.554	*V* = 318.34 ± 0.686
	*P* = 0.468 ± 0.003	***P* = 0.0005 ± 0.00004**	***P* = 0.0001 ± 0.000008**	*P* = 0.24 ± 0.0027
BS/NBS	*V* = 160.45 ± 0.539	*V* = 288.8 ± 0.667	*V* = 593.8 ± 0.592	*V* = 318.15 ± 0.699
	*P* = 0.023 ± 0.0006	*P* = 0.355 ± 0.003	***P* = 0.00006 ± 0.000006**	*P* = 0.106 ± 0.0018
PCS/NBS	*V* = 114.18 ± 0.455	*V* = 601.19 ± 0.528	*V* = 276.93 ± 0.663	*V* = 203 ± 0.591
	***P* = 0.003 ± 0.0002**	***P* = 0.006 ± 0.528**	*P* = 0.487 ± 0.003	*P* = 0. 416 ± 0.003

Paired Wilcoxon signed-rank test, α = 0.017, significant differences in bold, nonparametric bootstrap *N* = 38, 10,000 iterations, mean ± SE.

BS, breeding season; NBS, nonbreeder season; PCS, parental care season.

The relative number of days that individuals across the different presence categories were present was significantly different between seasons for *resident* birds and *continuous* visitors (Approximative Friedman test, 10,000 Monte-Carlo iterations; *resident* birds: *N* = 26, *χ*^*2*^ = 17.146, *P* < 0.001; *continuous* visitors: *N* = 41, *χ*^*2*^ = 19.858, *P* < 0.001; *periodic* visitors: *N* = 15, *χ*^*2*^ = 3.444, *P* = 0.178; *rare* visitors: *N* = 47, *χ*^*2*^ = 3.475, *P* = 0.172). Breeding season differed significantly in *resident* birds and parental care season differed significantly in *continuous* visitors (exact paired Wilcoxon signed-rank tests; *Resident* Birds, *N* = 26: Nonbreeder Season ~ Breeding Season, *Z* = 3.318, *P* < 0.001, Parental Care Season ~ Breeding Season, *Z* = 2.666, *P* = 0.003, Nonbreeder Season ~ Breeding Season, *Z* = 0.102, *P* = 0.925; *Continuous* Visitors, *N* = 41: Nonbreeder Season ~ Breeding Season, *Z* = 1.067, *P* = 0.291, Parental Care Season ~ Breeding Season, *Z* = 3.411, *P* < 0.001, Nonbreeder Season ~ Breeding Season, *Z* = −3.069, *P* < 0.001). Specifically, the *resident* birds were seen significantly less during breeding season (mean ± SD; breeding season: 13.03 ± 5.92, parental care season: 19.23 ± 6.57, nonbreeder season: 19.15 ± 7.27) and *continuous* visitors were seen significantly more often during parental care season (mean ± SD; breeding season: 3.71 ± 3.12, parental care season: 7.85 ± 5.03, nonbreeder season: 5.76 ± 4.49).

### Environmental influences on local flock size

In the final model that was derived from the weighted average of all models, the factors with the strongest effect on local flock size were: transect time, then season, temperature, and weather (prediction 1 and 3, Table 2).

**Table 2:** Influences on local flock size, average model

		Estimate ± SE	Pr(>|z|)
	(Intercept)	4.007 ± 0.052	<0.001
Transect Time	Noon	−0.020 ± 0.006	0.001
	Afternoon	0.534 ± 0.004	<0.001
Season	Breeding Season	−0.303 ± 0.073	<0.001
	Parental care season	0.146 ± 0.072	0.042
Temperature	Warm	−0.285 ± 0.014	<0.001
	Hot	−0.189 ± 0.020	<0.001
Weather	Clouds	0.154 ± 0.011	<0.001
	Sun	0.177 ± 0.012	<0.001
Risk of Enclosure	High Risk	0.031 ± 0.007	<0.001
Visitors	Some	0.004 ± 0.004	0.391
	Many	0.027 ± 0.012	0.018

Calculated from all models (weighted full average), the factors are ordered by influence from high to low and by their category.

### Subgroup size

**Figure 4:**
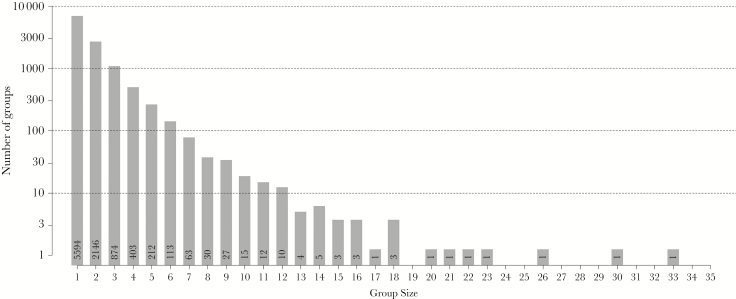
Histogram of the observed subgroup sizes; *y* axis log-transformed. Numbers in bars show the exact number of observed subgroups.

Subgroup size, as determined by our definition of all individuals within 5 m of one another with a direct line of sight to at least one other crow, was found to range from 1 up to 33 individuals (Figure 4). The mean subgroup size ± SD was 1.85 ± 1.64, *N* = 9525 (Quantiles: 0%: 1; 25%: 1; 50%: 1; 75%: 2; 100%: 33). When considering only subgroups of 2 individuals or more, the mean subgroup size ± SD was 3.07 ± 2.00, *N* = 3931 (Quantiles: 0%: 2; 25%: 2; 50%: 2; 75%: 3; 100%: 33). Note that the subgroup sizes differed between the 4 presence categories (Kruskal–Wallis test, *H*_3_ = 51.222, *P* < 0.001). However, when correcting for multiple pairwise comparisons by setting α to 0.0083, the only statistical significance remained for *resident* birds. These birds were found in smaller subgroups compared with the birds in other presence categories; *continuous* and *periodic* visitors did not differ significantly in subgroup size (Table 3).

**Table 3:** Pairwise comparisons for the subgroup sizes across the 4 presence categories

	*N*	Mean ± SD	Resident birds	Continuous visitors	Periodic visitors
Resident Birds	1754	2.46 ± 2.91			
Continuous Visitors	779	3.34 ± 4.56	W = 585,130 ***P* < 0.001**		
Periodic Visitors	183	2.96 ± 3.30	W = 136,960 ***P* < 0.001**	W = 71,442 *P* = 0.960	
Rare Visitors	88	3.66 ± 5.03	W = 59,463 ***P* < 0.001**	W = 31,384 *P* = 0.181	W = 7331 *P* = 0.219

Wilcoxon signed-rank tests, α = 0.008.

### Influences on subgroup size

We derived the final model by averaging all models. We found that all factors had a significant effect on subgroup size (prediction 4, Table 4).

**Table 4:** Influences on subgroup size, average model

		Estimate ± SE	Pr(>|z|)
	(Intercept)	−0.549 ± 0.152	<0.001
Season	Breeding Season	−0.633 ± 0.102	<0.001
	Parental Care Season	−0.169 ± 0.094	0.071
Forested Area	Out of Forest	0.605 ± 0.034	<0.001
On Building	Off	0.489 ± 0.040	<0.001
Age	Nonjuvenile	−0.451 ± 0.032	<0.001
Food	Vegetarian	0.423 ± 0.024	<0.001
	Mixed	−0.034 ± 0.047	0.473
	Mainly Meat	0.263 ± 0.039	<0.001
	Human Gastronomical Area	−0.291 ± 0.044	<0.001
Weather	Clouds	0.317 ± 0.065	<0.001
	Sun	0.138 ± 0.067	0.040
Transect Time	Noon	−0.113 ± 0.035	0.002
	Afternoon	0.280 ± 0.025	<0.001
Visitors	Some	−0.099 ± 0.026	<0.001
	Many	0.195 ± 0.055	<0.001
Number of Crows Present	0.005 ± 0.002	0.009

Calculated from all models (weighted full average), the factors are ordered by influence from high to low and by their category.

## DISCUSSION

Our findings support the majority of our hypotheses regarding grouping behavior and the influence of environmental factors on population, group size and their composition with regard to residency status (presence categories) in wild carrion/hooded crows utilizing Vienna Zoo. The local flock’s size changed considerably throughout the year and was significantly smaller during the breeding season than other seasons (prediction 1). The composition of the crow local flock resembled that of wild common ravens (Braun and Bugnyar 2012), and consisted of resident birds and birds that visit the area either continuously, periodically or only rarely (prediction 2). Environmental factors such as time of day, season, temperature, and weather had a significant effect on the size of the local flock (prediction 3). There were also significant environmental effects on subgroup size (prediction 4), in particular season, structure of the surroundings, age class of the birds, and weather. Relating these findings to the framework for fission–fusion dynamics proposed by Aureli et al. (2008), temporal variation in party size is evident on the flock and subgroup level, whereas hints towards a temporal variation in party composition are found on the level of the local flock with regard to changes in the residency status of birds.

### Crow social structure and dynamics

From a socio-cognitive perspective, the structure of the local flock is interesting. Our data fit the well-known picture of corvids forming “open” groups when utilizing resources, with individuals coming and going at different times and rates (Coombs 1978; Richner 1989b; Marzluff and Heinrich 1991). Further, they corroborate recent findings in ravens that such “groups” (termed here as the local flock) are composed of individuals with different degrees of residency and vagrancy, respectively, with some individuals showing stronger preferences for a given site than others (Braun and Bugnyar 2012; Loretto et al. 2016). Moreover, our current analyses of the presence patterns show that a more detailed differentiation is possible: in addition to “resident” birds staying in the Zoo and “continuous visitors” coming to the Zoo regularly, we can distinguish “periodic visitors” coming to the Zoo only for certain time periods from “rare visitors” only rarely being sighted within the Zoo area. From the first 3 categories, some crows could be identified through observations as breeders. The majority of birds in all categories, however, seems to be nonbreeders that are either too young to breed or that failed to find a partner and/or defend a breeding territory.

The proportion of individuals belonging to the 4 presence categories underwent significant changes throughout the year. The proportion of resident birds was higher during the nonbreeder season, which could be due to food availability or to decreased competition for breeding sites in fall and winter. The lower proportion of birds during the breeding season could also be due to resident breeders being less visible while present at the nest. Indeed, the relative number of days that resident individuals were seen is significantly lower during the breeding season. The proportion of continuous visitors was significantly higher during the parental care season than the other seasons, which could stem from small family groups preferably living in the areas surrounding the Zoo, though coming in to the Zoo to raise their young in an area with higher food density. While resident birds and continuous visitors were more similar according to the cluster analysis, periodic visitors were more distinct regarding their presence. The proportion of periodic visitors in the population was higher during the breeding season than the rest of the year. Therefore, we assume birds within this category may come to the Zoo to attempt breeding as the area presents a secure feeding site.

Rare visitors were sighted evenly across all seasons. Therefore, we assume that these individuals could be vagrant birds using large areas, similar to previous findings in ravens (Loretto et al. 2016). They may use the Zoo only as a stopover during vagrancy, or the Zoo may be situated at the periphery of their home ranges and the birds seldom visit the area. During observations, we did not observe all the individuals that were marked as part of the longer-term project. This could in part be due to death, as indicated in Figure 3 where 3 individuals were known to have died during the period of the present study, albeit not all in the Zoo. However, we found evidence that some individuals that were marked at the Zoo seemed to have left the area for several months before being sighted there again. This is particularly apparent in the “Rare Visitors” and “Periodic Visitors” category. Therefore, it is plausible that a certain proportion of birds only return irregularly to our study area and some individuals may not return to the Zoo at all.

Although our categories are constructs that describe the crows’ presence patterns along a continuum, it is worth noting that residents and continuous visitors should differ in their likelihood of meeting one another, when compared with periodic or rare visitors. Hence, the different presence patterns may have direct effects on the birds’ social knowledge and behavior. For instance, the birds with spatio-temporally stable patterns may come to recognize each other in individual terms, whereas those with high degrees of vagrancy may be treated according to rules of thumb, such as always supporting the aggressor in agonistic interactions. Further studies are needed to test such assumptions.

Overall, the number of crows using the Zoo differed significantly between the breeding season and the rest of the year, with fewer birds being present during the breeding season, which is consistent with our prediction. This finding could be explained by the territoriality of breeding pairs trying to defend food resources needed for their offspring, similar to American crows (Marzluff and Heinrich 1991; Webb et al. 2012). As breeding territories cover most of the Zoo area, breeding pairs could possibly repel nonbreeders from foraging there. A study on the same local flock found the crows to be highly tolerant towards other individuals foraging in the same area even in the late breeding season and parental care season, which was likely due to the high availability of food (Miller et al. 2014). However, this study did not investigate any seasonal effects on tolerance and measured tolerance via social interactions, rather than our present measures of local flock and subgroup size. Therefore, it is possible that the high tolerance between crows found in the Miller et al. (2014) study may be habitat/population specific. Alternatively, it could be a “dear enemy” effect (Ydenberg et al. 1988), where individuals sharing territories close to one another allow foraging in close vicinity. This tolerance towards conspecifics could be a common trait among corvid species. Studies on social networks in New Caledonian Crows (*Corvus moneduloides*) and ravens show that information flow within groups is fast and flexible, and can be predicted by association patterns within groups but less so between groups that are less closely associated spatially (St Clair et al. 2015; Kulahci et al. 2016). Future studies could investigate seasonal differences and habitat specific differences on tolerance in a foraging context within other carrion/hooded crow populations. In regard to the present study, despite the generally high number of crows present and ensuing high competition, the high availability of food in the Zoo may explain why breeding pairs appeared to defend their territories during the breeding season only.

### Environmental effects

Our measures of local flock size and subgroup size are likely linked, as a larger local flock size could increase the possibility of larger subgroups forming, as indicated in sea birds (Beauchamp 2011). Hence, the environmental factors that influenced the local flock size affected subgroup size in a similar manner. The crows’ activity patterns are strongly influenced by the time of day, weather and temperature. Hot temperatures (>25 °C) and rainy weather likely lowered the crows’ mobility, leading to smaller local flock and subgroup sizes as fewer crows with territories outside of the Zoo congregated within the Zoo area. Local flock and subgroup size seem to have a strong link to the availability of food in the Zoo, as they increase significantly in times when food availability in the Zoo is likely to be higher than outside this area. For instance, food available may be higher within the Zoo in colder mean temperature parts of the year (local flock size only), as well as in the afternoon when the birds may have a higher chance of stealing food from animal enclosures, and feeding on the food dropped by visitors, similar to foraging strategies of habituated grizzly bears (Albert and Bowyer 1991). The relative abundance of food appears to be a strong social facilitator in terms of both subgroup size and local flock size, similar to ravens converging on carcasses during winter months (Heinrich 1989).

Forest cover, habitat openness and man-made structures influenced subgroup size (prediction 4). Large subgroups of crows were more likely to form in the more open, nonforested part of the zoo. This may be due to larger subgroups providing more protection from potential predation, which is more likely to occur in open areas (Jarman 1974), or potential risk from humans. We also consider potential sampling limitations with lower visibility in forested areas during the summertime. Foliage may prevent crows that were sitting in trees from being observed, therefore lowering the estimate for the population or subgroup size, while better visibility during winter may lead to higher estimates in either measure. However, a study in another population of hooded crows also found a preference for nesting in more open areas (Kövér et al. 2015), suggesting that there may be a general preference for crows to make use of open areas.

Alternatively, the availability and distribution of food in relation to habitat structure may play an important role. Availability of food was generally high as the crows had access both to food provided for zoo animals as well as food dropped or fed to the crows by visitors. Despite this generally high availability of food, the largest recorded subgroup (*n* = 33) was found in an enclosure with widely scattered food (black crowned crane, *Balearica pavonia*). This wide scattering of food likely allowed for a large subgroup of birds to forage and feed together, while lowering the chances of conflicts, similar to findings in spider monkeys (Asensio et al. 2008, 2009). In support of this explanation, subgroup size tended to be larger in the mainly “vegetarian” animal enclosures of the zoo, where food is generally scattered across larger areas compared with enclosures housing carnivorous predator species, where meat/fish is usually presented in a concentrated manner. Similar results have been found in white-throated magpies, where higher food quantity allowed for larger foraging parties (Langen and Vehrencamp 1998).

The number of human visitors present at the Zoo has a significant effect on subgroup size, where a high number of human visitors is associated with an increase in subgroup size. It is possible that crows avoid crowds of visitors in the public areas by preferentially spending time within animal enclosures, thereby increasing the likelihood for subgroup formation. Another influential factor for subgroup size was the age class of the crows. Similar to other corvids (Goodwin 1976), nonjuvenile crows were less likely to form subgroups, as they tended to have formed pair bonds and engaged in territorial behavior compared with juveniles.

In summary, our findings suggest that carrion and hooded crows in our local flock showed fission–fusion dynamics. The local flock and its subgroup dynamics were influenced by environmental factors. These findings have interesting implications for social complexity in these crows as, for instance, different areas in the Zoo appear to have positive or negative effects on subgroup size. In addition, we identified different levels of temporal flock membership that require further exploration into the potential relationships within and between the different presence categories of crows as well as implications on the information flow between birds. Further, future studies may aim to compare urban and rural populations of crows to explore whether our findings are habitat specific or applicable more generally to this species, as well as other avian species.

## FUNDING

This work was supported by the Vienna Science and Technology Fund (CS11-008 to C.S.); the Austrian Science Fund (Y366-B17 and W1234-G17 to T.B., J3868-B25 and P24788-B22 [PI: Eva Ringler] to M.R.); and by the Tiergarten Schönbrunn (Zoo Vienna).

## 

Data accessibility: Analysis in this article reproduced by using the original data set provided by Uhl et al. 2018.

## Supplementary Material

Supplementary Material

## References

[CIT0001] Albert DM, Bowyer RT 1991 Factors related to grizzly bear: human interactions in Denali National Park. Wildl Soc Bull. 19:339–349.

[CIT0002] Amici F, Aureli F, Call J 2008 Fission-fusion dynamics, behavioral flexibility, and inhibitory control in primates. Curr Biol. 18:1415–1419. 18804375 10.1016/j.cub.2008.08.020

[CIT0003] Anderson DR 2008 Model based inference in the life sciences: a primer on evidence. New York: Springer Science+Business Media, LLC.

[CIT0004] Anderson DR, Burnham KP 2002 Avoiding pitfalls when using information-theoretic methods. J Wildl Manage. 66:912–918.

[CIT0005] Asensio N, Korstjens AH, Aureli F 2009 Fissioning minimizes ranging costs in spider monkeys: a multiple-level approach. Behav Ecol Sociobiol. 63:649–659.

[CIT0006] Asensio N, Korstjens AH, Schaffner CM, Aureli F 2008 Intragroup aggression, fission-fusion dynamics and feeding competition in spider monkeys. Behaviour. 145:983–1001.

[CIT0007] Aureli F, Schaffner CM, Boesch C, Bearder SK, Call J, Chapman CA, Connor R, Di Fiore A, Dunbar RIM, Henzi SP, et al 2008 Fission-fusion dynamics: new research frameworks. Curr Anthropol. 49:627–654.

[CIT0008] Baglione V, Marcos J, Canestrari D, Murphy M 2002 Cooperatively breeding groups of carrion crow (*Corvus corone corone*) in northern Spain. Auk. 119:790–799.

[CIT0009] Barton K 2017 MuMIn: multi model inference. R package version 1.40.0. Available from: https://CRAN.R-project.org/package=MuMIn. Accessed 27 November 2017

[CIT0010] Bates D, Maechler M, Bolker B, Walker S 2015 Fitting linear mixed models using lme4. J Stat Softw. 67:1–48.

[CIT0011] Beauchamp G 2011 Functional relationship between group size and population density in Northwest Atlantic seabirds. Mar Ecol Prog Ser. 435:225–233.

[CIT0012] von Blotzheim GU 1993 Handbuch der Voegel Mitteleuropas. Wiesbaden: Aula-Verlag.

[CIT0013] Bond AB, Kamil AC, Balda RP 2003 Social complexity and transitive inference in corvids. Anim Behav. 65:479–487.

[CIT0014] Boucherie PH, Mariette MM, Bret C, Dufour V 2016 Bonding beyond the pair in a monogamous bird: impact on social structure in adult rooks (*Corvus frugilegus*). Behaviour. 153:897–925.

[CIT0015] Bradbury JW, Balsby TJS 2016 The functions of vocal learning in parrots. Behav Ecol Sociobiol. 70:293–312.

[CIT0016] Braun A, Bugnyar T 2012 Social bonds and rank acquisition in raven nonbreeder aggregations. Anim Behav. 84:1507–1515. 23264693 10.1016/j.anbehav.2012.09.024 PMC3518779

[CIT0017] Braun A, Walsdorff T, Fraser ON, Bugnyar T 2012 Socialized sub-groups in a temporary stable Raven flock? J Ornithol. 153:97–104. 25892747 10.1007/s10336-011-0810-2 PMC4398859

[CIT0018] Brown JL 1970 Cooperative breeding and altruistic behaviour in the Mexican jay, *Aphelocoma ultramarina*. Anim Behav. 18:366–378.

[CIT0019] Burnham KP, Anderson DR 2004 Multimodel inference. Sociol Methods Res. 33:261–304.

[CIT0020] Caraco T, Wolf LL 1975 Ecological determinants of group sizes of foraging lions. Am Nat. 109:343–352.

[CIT0021] Chapman CA, Pavelka MS 2005 Group size in folivorous primates: ecological constraints and the possible influence of social factors. Primates. 46:1–9. 15197599 10.1007/s10329-004-0093-9

[CIT0022] Chapman CA, Wrangham RW, Chapman LJ 1995 Ecological constraints on group size : an analysis of spider monkey and chimpanzee subgroups. Behav Ecol Sociobiol. 36:59–70.

[CIT0023] Clayton NS, Emery NJ 2007 The social life of corvids. Curr Biol. 17:R652–R656. 17714658 10.1016/j.cub.2007.05.070

[CIT0024] Cockburn A 2006 Prevalence of different modes of parental care in birds. Proc Biol Sci. 273:1375–1383. 16777726 10.1098/rspb.2005.3458 PMC1560291

[CIT0025] Coombs F 1978 The crows: a study of the corvids of Europe. In: The crows: a study of the corvids of Europe. London (UK): BT Batsford Limited.

[CIT0026] Cords M, Aureli F 2000 Reconciliation and relationship qualities. In: Aureli F, De Waal FBM, editors. Natural conflict resolution. Berkeley and Los Angeles: University of California Press p. 177–198.

[CIT0027] Deventer SA, Uhl F, Bugnyar T, Miller R, Fitch WT, Schiestl M, Ringler M, Schwab C 2016 Behavioural type affects space use in a wild population of crows (*Corvus corone*). Ethology. 122:881–891. 27840464 10.1111/eth.12536 PMC5082553

[CIT0028] Emery NJ, Seed AM, von Bayern AM, Clayton NS 2007 Cognitive adaptations of social bonding in birds. Philos Trans R Soc Lond B Biol Sci. 362:489–505. 17255008 10.1098/rstb.2006.1991 PMC2346513

[CIT0029] Goodwin D 1976 Crows of the world. 1st ed. London: The British Museum (Natural History).

[CIT0030] Hamilton WD 1971 Geometry for the selfish herd. J Theor Biol. 31:295–311. 5104951 10.1016/0022-5193(71)90189-5

[CIT0031] Harcourt AH, de Waal FBM 1992 Coalitions and alliances in humans and other animals. Oxford: Oxford University Press.

[CIT0032] Heinrich B 1989 Ravens in winter. New York: Summit Books of Simon & Schuster.

[CIT0033] Heinrich B, Kaye D, Knight T, Schaumburg K 1994 Dispersal and association among common ravens. Condor. 96:545–551.

[CIT0034] Hobson EA, Avery ML, Wright TF 2014 The socioecology of monk parakeets: insights into parrot social complexity. Auk. 131:756–775.

[CIT0035] Hothorn T, Hornik K, van de Wiel MA, Zeileis A 2008 Implementing a class of permutation tests: the coin package. J Stat Softw. 28:1–23.27774042

[CIT0036] Jarman PJ 1974 The social organisation of antelope in relation to their ecology. Behaviour. 48:215–267.

[CIT0037] Kappeler PM, van Schaik CP 2002 Evolution of primate social systems. Int J Primatol. 23:707–740.

[CIT0039] de Knijff P 2014 How carrion and hooded crows defeat Linnaeus’s curse. Science. 344:1345–1346. 24948724 10.1126/science.1255744

[CIT0040] Kövér L, Gyüre P, Balogh P, Huettmann F, Lengyel S, Juhász L 2015 Recent colonization and nest site selection of the Hooded Crow (*Corvus corone cornix* L.) in an urban environment. Landsc Urban Plan. 133:78–86.

[CIT0041] Krause J, Ruxton GD 2002 Living in groups. Oxford: Oxford University Press.

[CIT0042] Kulahci IG, Rubenstein DI, Bugnyar T, Hoppitt W, Mikus N, Schwab C 2016 Social networks predict selective observation and information spread in ravens. R Soc Open Sci. 3:160256. 27493780 10.1098/rsos.160256 PMC4968472

[CIT2325] Kummer H 1971 Primate Societies: group techniques of ecological adaptation. Chicago (IL): Aldine Publishing Company.

[CIT0043] Kurvers RHJM, Adamczyk VMAP, Kraus RHS, Hoffman JI, van Wieren SE, van der Jeugd HP, Amos W, Prins HHT, Jonker RM 2013 Contrasting context dependence of familiarity and kinship in animal social networks. Anim Behav. 86:993–1001.

[CIT0044] Langen TA, Vehrencamp SL 1998 Ecological factors affecting group and territory size in white-throated magpie-jays. Auk. 115:327–339.

[CIT0045] Loretto M-C, Reimann S, Schuster R, Graulich DM, Bugnyar T 2015 Shared space, individually used: spatial behaviour of non-breeding ravens (*Corvus corax*) close to a permanent anthropogenic food source. J Ornithol. 157:1–12.

[CIT0046] Loretto MC, Schuster R, Bugnyar T 2016 GPS tracking of non-breeding ravens reveals the importance of anthropogenic food sources during their dispersal in the Eastern Alps. Curr Zool. 62:337–344. 29491922 10.1093/cz/zow016 PMC5829441

[CIT0047] Loretto MC, Schuster R, Itty C, Marchand P, Genero F, Bugnyar T 2017 Fission-fusion dynamics over large distances in raven non-breeders. Sci Rep. 7:380. 28336913 10.1038/s41598-017-00404-4 PMC5428508

[CIT0048] Marra PP, Cohen EB, Loss SR, Rutter JE, Tonra CM 2015 A call for full annual cycle research in animal ecology. Biol Lett. 11:20150552. 26246337 10.1098/rsbl.2015.0552 PMC4571685

[CIT0049] Marzluff JM, Angell T 2005 In the company of crows and ravens. New Haven (CT): Yale University Press.

[CIT0050] Marzluff JM, Heinrich B 1991 Foraging by common ravens in the presence and absence of territory holders: an experimental analysis of social foraging. Anim Behav. 42:755–770.

[CIT0051] Massen JJ, Pašukonis A, Schmidt J, Bugnyar T 2014 Ravens notice dominance reversals among conspecifics within and outside their social group. Nat Commun. 5:3679. 24755739 10.1038/ncomms4679 PMC3997804

[CIT0052] Miller R, Schiestl M, Whiten A, Schwab C, Bugnyar T 2014 Tolerance and social facilitation in the foraging behaviour of free-ranging crows (*Corvus corone corone*; C. c. cornix). Ethology. 120:1248–1255. 25937686 10.1111/eth.12298 PMC4415146

[CIT0053] Paz-y-Mino G, Bond AB, Kamil AC, Balda RP 2004 Pinyon jays use transitive inference to predict social dominance. Nature. 430:778–781. 15306809 10.1038/nature02723

[CIT0054] Peek JM, LeResche RE, Stevens DR 1974 Dynamics of moose aggregations in Alaska, Minnesota, and Montana. J Mammal. 55:126–137.

[CIT0055] Poelstra JW, Vijay N, Bossu CM, Lantz H, Ryll B, Müller I, Baglione V, Unneberg P, Wikelski M, Grabherr MG, et al 2014 The genomic landscape underlying phenotypic integrity in the face of gene flow in crows. Science. 344:1410–1414. 24948738 10.1126/science.1253226

[CIT0056] Pradel R, Hines JE, Lebreton J, Nichols JD 1997 Capture-recapture survival models taking account of transients. Biometrics. 53:60.

[CIT0057] Randler C 2007 Assortative mating of carrion *Corvus corone* and hooded crows *C. cornix* in the Hybrid Zone in Eastern Germany. Ardea. 95:143–149.

[CIT0058] Randler C 2008 Mating patterns in avian hybrid zones - a meta-analysis and review. Ardea. 96:73–80.

[CIT0059] R Core Team 2017 R: a language and environment for statistical computing. Version 3.4.2. Vienna (Austria): R Foundation for Statistical Computing. Available from: https://www.R-project.org/. Accessed 28 September 2017.

[CIT0060] Richner H 1989a Habitat-specific growth and fitness in carrion crows (*Corvus corone corone*). J Anim Ecol. 58:427–440.

[CIT0061] Richner H 1989b Phenotypic correlates of dominance in carrion crows and their effects on access to food. Anim Behav. 38:606–612.

[CIT0062] Risch M, Andersen L 1998 Selektive partnerwahl der aaskrähe (*Corvus corone*) in der hybridisierungszone von rabenkrähe (*C. c. corone*) und nebelkrähe (*C. c. cornix*). J Ornithol. 139:173–177.

[CIT0063] Scheiber IBR, Kotrschal K, Weiß BM, Hemetsberger J 2013 The social life of greylag geese. Cambridge: Cambridge University Press.

[CIT0064] Scheiber IB, Weiß BM, Frigerio D, Kotrschal K 2005 Active and passive social support in families of greylag geese (*Anser anser*). Behaviour. 142:1535–1557. 21984839 10.1163/156853905774831873 PMC3188404

[CIT0065] Silk MJ, Croft DP, Tregenza T, Bearhop S 2014 The importance of fission – fusion social group dynamics in birds. Ibis (Lond. 1859). 156:701–715.

[CIT0066] Smith JE, Kolowski JM, Graham KE, Dawes SE, Holekamp KE 2008 Social and ecological determinants of fission–fusion dynamics in the spotted hyaena. Anim Behav. 76:619–636.

[CIT0067] Smolker RA, Richards AF, Connor RC, Pepper JW 1992 Sex differences in patterns of association among Indian Ocean bottlenose dolphins. Behaviour. 123:38–69.

[CIT0068] St Clair JJ, Burns ZT, Bettaney EM, Morrissey MB, Otis B, Ryder TB, Fleischer RC, James R, Rutz C 2015 Experimental resource pulses influence social-network dynamics and the potential for information flow in tool-using crows. Nat Commun. 6:7197. 26529116 10.1038/ncomms8197 PMC4659832

[CIT0069] Stouffer PC, Caccamise DF 1991 Roosting and diurnal movements of radio-tagged American crows. Wilson Bull. 103:387–400.

[CIT0070] Szipl G, Ringler E, Spreafico M, Bugnyar T 2017 Calls during agonistic interactions vary with arousal and raise audience attention in ravens. Front Zool. 14:57. 29299036 10.1186/s12983-017-0244-7 PMC5740903

[CIT0071] Thirgood SJ 1996 Ecological factors influencing sexual segregation and group size in fallow deer (*Dama dama*). J Zool. 239:783–797.

[CIT0072] Thomas L, Buckland ST, Burnham KP, Anderson DR, Laake JL, Borchers DL, Strindberg S 2002 Distance sampling. In: ElShaarawi AH, Piegorschs WW, editors. Encyclopedia of environmetrics. Chichester (UK): John Wiley & Sons, Ltd.

[CIT0073] Treisman M 1975 Predation and the evolution of gregariousness. II. An economic model for predator-prey interaction. Anim Behav. 23:801–825.

[CIT0074] Uhl F, Ringler M, Miller R, Deventer S, Bugnyar T, Schwab C 2018 Data from: counting crows: flock structure and subgroup size variation in an urban population of crows. Dryad Digital Repository. 10.5061/dryad.t0g149j. PMC6398430 30846892

[CIT0075] Vander Wall SB, Balda RP 1977 Coadaptations of the Clark’s Nutcracker and the pinon pine for efficient seed harvest and dispersal. Ecol Monogr. 47:89–111.

[CIT0076] Wanker R 1999 Socialization in spectacled parrotlets (*Forpus conspicillatus*): how juveniles compensate for the lack of siblings. Acta Ethol. 2:23–28.

[CIT0077] Webb WC, Marzluff JM, Hepinstall-Cymerman J 2012 Differences in space use by common ravens in relation to sex, breeding status, and kinship. Condor. 114:584–594.

[CIT0078] Wolf JBW, Mawdsley D, Trillmich F, James R 2007 Social structure in a colonial mammal: unravelling hidden structural layers and their foundations by network analysis. Anim Behav. 74:1293–1302.

[CIT0079] Woolfenden GE, Fitzpatrick JW 1984 The Florida scrub jay: demography of a cooperative-breeding bird. Princeton (NJ): Princeton University Press.

[CIT0080] Ydenberg RC, Giraldeau LA, Falls JB 1988 Neighbours, strangers, and the asymmetric war of attrition. Anim Behav. 36:343–347.

