# Supplementary material for: Counting crows: population structure and group size variation in an urban population of crows
Source: Behav Ecol. 2018 Dec 8;30(1):57–67. doi: 10.1093/beheco/ary157 (PMC6398430; doi:10.1093/beheco/ary157)
Supplement: Supplementary Material [file ary157_suppl_supplementary_material.doc]

SUPPLEMENTARY DATA

Table S1. Parameters collected per individual crow in the field. Categories of parameters are given in bold letters, single parameters within the respective category in regular letters.

| ***Category*** | ***Parameter*** | ***Definition*** |
| --- | --- | --- |
| **General Info** | |  |
| **Date** |  | Date of the observation |
| **Time** |  | Time of the observation |
| **Observer** |  | Initials of the observer |
| **Temperature** | Cold | <5°C |
|  | Warm | 5-25°C |
|  | Hot | >25°C |
| **Weather** | Sun | Sun shines, few if any clouds |
|  | Clouds | Heavily overcast skies, also foggy days |
|  | Precipitation | Rain/Snow |
| **Visitors** | Many | Perceived amount of visitors present. |
|  | Some |
|  | Few |
| **Crow Parameters** | |  |
| **Marking** | Marked/Unmarked | Ringed crows are entered as "marked", Unringed crows as “unmarked” |
| **Age Class** | Juvenile | Young birds, still red in their beaks, first plumage |
|  | Sub-adults | Birds that are not juvenile any more, but still have their juvenile flight feathers, may be still red in their beaks |
|  | Adults | Birds with adult plumage |
| **Individual** | ID | If ID-Number of a bird is known to the observer, then it can be entered into a special box. |
|  |  |  |

Table S2. Information on the surroundings of each sighting added to the parameters in ArcMap. Note that each sighting contains information on all categories.

| **Category** | **Parameter** | **Description** |
| --- | --- | --- |
| **Forested Area** | In forest | Forested area of the Zoo |
|  | Out of forest | Non-forested area of the Zoo |
| **On Building** | On | Rooftop or man-made structure that cannot be accessed by visitors |
|  | Off | On the floor or on trees |
| **Food** | Grass | Only grass fed to animals in enclosure |
|  | Vegetarian | Grass and Fruits fed to animals in enclosure |
|  | Mixed | Mainly vegetarian food fed to animals, but also meat |
|  | Mainly Meat | Mainly meat fed to animals in enclosure |
|  | Human Gastronomical Area | Locations with gastronomical facilities for zoo visitors |

Table S3. Correlation matrix for the flock size estimate. I is the Intercept. This is derived from the full model without model averaging.

|  | I | W C | WS | T W | T H | V S | V M | T T N | T T A | S B S |  |  |  |  |  |  |  |  |
| --- | --- | --- | --- | --- | --- | --- | --- | --- | --- | --- | --- | --- | --- | --- | --- | --- | --- | --- |
| Weather Clouds | -0.208 |  |  |  |  |  |  |  |  |  |  |  |  |  |  |  |  |  |
| Weather Sun | -0.203 | 0.872 |  |  |  |  |  |  |  |  |  |  |  |  |  |  |  |  |
| Temperature Warm | -0.175 | 0.106 | 0.115 |  |  |  |  |  |  |  |  |  |  |  |  |  |  |  |
| Temperature Hot | -0.124 | 0.073 | 0.081 | 0.747 |  |  |  |  |  |  |  |  |  |  |  |  |  |  |
| Visitors Some | 0.008 | -0.078 | -0.087 | 0.051 | -0.03 |  |  |  |  |  |  |  |  |  |  |  |  |  |
| Visitors Many | 0.004 | -0.035 | -0.029 | 0.022 | -0.059 | 0.318 |  |  |  |  |  |  |  |  |  |  |  |  |
| Transect Time Noon | -0.009 | 0.028 | 0.019 | -0.14 | -0.197 | -0.256 | -0.217 |  |  |  |  |  |  |  |  |  |  |  |
| Transect Time Afternoon | -0.013 | 0.046 | 0.014 | -0.263 | -0.289 | -0.204 | -0.085 | 0.528 |  |  |  |  |  |  |  |  |  |  |
| Season Breeding Season | -0.646 | -0.006 | -0.017 | -0.063 | -0.045 | -0.019 | -0.015 | 0.007 | 0.023 |  |  |  |  |  |  |  |  |  |
| Season Parental Care Season | -0.656 | -0.011 | -0.021 | -0.08 | -0.067 | -0.021 | -0.011 | 0.003 | 0.023 | 0.481 |  |  |  |  |  |  |  |  |

Table S4. Correlation matrix for the subgroup size. I is the Intercept. This is derived from the full model without model averaging.

|  | I | W C | W S | T W | T H | V S | V M | A NJ | TT N | TT A | S B S | S P C S | FA OOF | F V | F M | F MM | F HGA | OB O |
| --- | --- | --- | --- | --- | --- | --- | --- | --- | --- | --- | --- | --- | --- | --- | --- | --- | --- | --- |
| Weather Clouds | -0.351 |  |  |  |  |  |  |  |  |  |  |  |  |  |  |  |  |  |
| Weather Sun | -0.316 | 0.876 |  |  |  |  |  |  |  |  |  |  |  |  |  |  |  |  |
| Temperature Warm | -0.234 | 0.115 | 0.102 |  |  |  |  |  |  |  |  |  |  |  |  |  |  |  |
| Temperature Hot | -0.149 | 0.079 | 0.065 | 0.696 |  |  |  |  |  |  |  |  |  |  |  |  |  |  |
| Visitors Some | 0.038 | -0.093 | -0.105 | 0.034 | -0.055 |  |  |  |  |  |  |  |  |  |  |  |  |  |
| Visitors Many | 0.019 | -0.05 | -0.028 | 0.017 | -0.096 | 0.324 |  |  |  |  |  |  |  |  |  |  |  |  |
| Age Non-Juvenile | -0.225 | 0.01 | 0.034 | -0.006 | -0.005 | 0.05 | -0.008 |  |  |  |  |  |  |  |  |  |  |  |
| Transect Time Noon | -0.05 | 0.03 | 0.032 | -0.134 | -0.186 | -0.266 | -0.205 | 0.022 |  |  |  |  |  |  |  |  |  |  |
| Transect Time Afternoon | -0.038 | 0.05 | 0.039 | -0.238 | -0.268 | -0.191 | -0.094 | 0.015 | 0.511 |  |  |  |  |  |  |  |  |  |
| Season Breeding Season | -0.377 | -0.063 | -0.112 | -0.26 | -0.17 | -0.084 | -0.066 | -0.015 | 0.035 | 0.074 |  |  |  |  |  |  |  |  |
| Season Parental Care Season | -0.285 | -0.065 | -0.096 | -0.312 | -0.251 | -0.084 | -0.05 | 0.073 | 0.016 | 0.095 | 0.528 |  |  |  |  |  |  |  |
| Forested Area Out of Forest | -0.218 | 0.014 | 0.003 | -0.011 | -0.001 | -0.039 | -0.017 | -0.006 | 0.034 | -0.023 | 0.016 | -0.016 |  |  |  |  |  |  |
| Food Vegetarian | 0.038 | 0.032 | 0.048 | -0.061 | -0.057 | -0.021 | -0.098 | 0.026 | -0.028 | 0.047 | 0.014 | 0.008 | -0.191 |  |  |  |  |  |
| Food Mixed | 0 | 0 | 0.016 | -0.025 | -0.024 | 0.042 | -0.004 | 0.042 | 0.061 | 0.102 | 0.015 | 0.013 | -0.107 | 0.181 |  |  |  |  |
| Food Mainly Meat | -0.018 | -0.035 | -0.024 | -0.058 | -0.047 | 0.033 | -0.006 | 0.007 | -0.028 | 0.037 | 0.026 | 0.032 | 0.095 | 0.161 | 0.087 |  |  |  |
| Food Human Gastronomical Area | 0.014 | 0.007 | 0.01 | 0.007 | 0.017 | -0.014 | -0.05 | 0.017 | -0.023 | -0.053 | -0.001 | -0.009 | -0.081 | 0.186 | 0.079 | 0.089 |  |  |
| On Building Off Building | -0.247 | -0.05 | -0.066 | 0.009 | 0.015 | -0.024 | -0.008 | 0.02 | -0.036 | -0.015 | 0.006 | 0.02 | 0.09 | -0.189 | -0.107 | -0.096 | -0.099 |  |
| Crows Present | -0.638 | -0.092 | -0.122 | -0.109 | -0.079 | -0.011 | 0.012 | -0.021 | 0.026 | -0.022 | 0.321 | 0.16 | 0 | 0 | 0.005 | 0.021 | 0.006 | -0.006 |

Table S5. The temporal autocorrelation structure for the flock size estimate. Calculated in R via acf().

| Lag | ACF |
| --- | --- |
| 1 | 0.235 |
| 2 | 0.257 |
| 3 | 0.223 |
| 4 | 0.31 |
| 5 | 0.219 |
| 6 | 0.125 |
| 7 | 0.153 |
| 8 | 0.145 |
| 9 | 0.064 |
| 10 | 0.046 |
| 11 | 0.096 |
| 12 | 0.032 |
| 13 | -0.001 |
| 14 | 0.014 |
| 15 | -0.038 |

Table S6. Summary of the four presence categories. The measure for absence was the number of observation days in which a crow was not seen.

| **Category** | **No. of Individuals** | **Mean Absence (±SD)** | | **Mean Days Observed (±SD)** | |
| --- | --- | --- | --- | --- | --- |
| **Resident Birds** | 26 | 6.75 | (± 6.78) | 54.19 | (± 23.28) |
| **Continuous Visitors** | 41 | 20.14 | (± 27.68) | 17.17 | (± 8.90) |
| **Periodic Visitors** | 15 | 31.80 | (± 63.44) | 11.13 | (± 6.71) |
| **Rare Visitors** | 47 | 126.22 | (± 108.85) | 2.72 | (± 1.12) |
